# Audit of the impact of the integrated psychological medicine service (IPMS) on service utilisation

**DOI:** 10.1192/bjo.2021.252

**Published:** 2021-06-18

**Authors:** Sarah Harvey, Joanna Bromley, Miles Edwards, Megan Hooper, Hannah McAndrew, Joanne Timms

**Affiliations:** 1 Devon Partnership Trust; 2 RD&E Hospital; 3 Exeter Medical School

## Abstract

**Aims:**

An audit to assess the impact of an Integrated Psychological Medicine Service (IPMS) on healthcare utilization pre & post intervention. We hypothesized that an IPMS approach would reduce healthcare utilization.

**Background:**

The IPMS focusses on integrating biopsychosocial assessments into physical healthcare pathways. It has developed in stages as opportunities presented in different specialities leading to a heterogeneous non-standardised service. The key aim is involvement of mental health practitioners, psychologists & psychiatrists in complex patients with comorbidity or functional presentations in combination with the specialty MDT. This audit is the first attempt to gather data across all involved specialities and complete a randomised deep dive into cases.

**Method:**

Referrals into IMPS from July 2019 to June 2020 pulled 129 referrals, of which a 10% randomised sample of 13 patients was selected to analyse. 5 patients had one year of data either side of the duration of the IPMS intervention (excluding 8 patients with incomplete data sets).

We analysed; the duration & nature of the IPMS intervention, the number, duration & speciality of inpatient admissions & short stays, outpatient attendances, non-attendances & patient cancellations. Psychosocial information was also gathered. One non-randomised patient was analysed as a comparative case illustration.

**Result:**

Randomised patients; patient 78's utilisation remained static, patient 71 post-referral engaged with health psychology & reduced healthcare utilisation. Patient 7 increased healthcare utilisation post-referral secondary to health complications. Patient 54 did not attend & increased healthcare utilisation post-referral. Patient 106 had increased healthcare utilisation post-referral from a new health condition. The randomised sample identified limitations of using healthcare utilisation as an outcome measure when contrasted to the non-randomised case (which significantly reduced healthcare utilisation post-referral).

**Conclusion:**

Correlation only can be inferred from the data due to sample size, limitations & confounding factors e.g. psycho-social life events, acquired illness. Alternative outcome measurements documented (e.g PHQ9/GAD7) were not reliably recorded across pathways.

The results evidenced that single cases can demonstrate highly desirable effects of a biopsychosocial approach but they can also skew data sets if results are pooled due to the small sample size & heterogeneous interventions. With some patients an increase in healthcare utilisation was appropriate for an improved clinical outcome. This audit identified that utilising healthcare utilisation as an outcome measure is a crude tool with significant limitations & the need to agree tailored outcome measures based on the type of intervention to assess the impact of IPMS.

